# Evaluating the implementation of a hospital-internal digital supply chain system over 5 years using a logic model framework

**DOI:** 10.3389/fdgth.2026.1772366

**Published:** 2026-07-30

**Authors:** Saskia Kröner, Jörg Hassmann, Lara Niemöller, Liling Wu, Birgit Babitsch, Ursula Hübner

**Affiliations:** 1 Centre for Medical, Social and Health Informatics, Osnabrück University of Applied Sciences (AS), Osnabrück, Lower Saxony, Germany; 2 New Public Health, Osnabrück University, Osnabrück, Lower Saxony, Germany

**Keywords:** evaluation, hospital digital transformation, implementation, logic model, mixed methods, satisfaction, supply chain, usage

## Abstract

**Introduction:**

Hospital digital transformation, including the integration of digital technologies into hospital supply chain management is critical for improving operational efficiency, workflow standardisation, and data quality in healthcare settings. This study evaluates the implementation of an internal supply chain project aimed at digitalising material documentation and request processes via an intelligent central hub and mobile barcode scanners in a hospital network.

**Methods:**

Employing a logic model framework, we conducted an embedded explanatory mixed-methods evaluation of a hospital digital transformation initiative over five years at Klinikum Region Hannover, Germany. Data sources included pre- and post-implementation user surveys (*n* = 310 and *n* = 148), anonymised system log data (2021–2025), and qualitative interviews with project leaders. The logic model mapped inputs, activities, outputs, and outcomes to assess evidence related to the proposed pathways of implementation success.

**Results:**

The implementation of the digital system accompanied by organisational measures achieved a transition from paper-based to scanner-based digital workflows, with increasing adoption rates, measurable process satisfaction and efficiency gains. Positive correlations between high process throughput and scanner usage suggest that higher process volumes encourage adoption, providing evidence for the proposed logic model pathway to adoption. A significant positive association was observed between satisfaction with staff contributions to the project and overall process satisfaction, providing pathway-consistent evidence for the proposed logic model pathway to satisfaction. Technical and organisational inputs enabled process re-engineering and workflow standardisation as outputs and time savings as outcomes, providing pathway-consistent evidence for the proposed logic model pathway to efficiency. Implementation outcomes varied across ten hospital sites, reflecting heterogenous local implementation conditions.

**Discussion:**

These findings confirm that comprehensive digital transformation of hospital supply chains can enhance satisfaction, workflow standardisation and efficiency but requires ongoing user engagement, continuous competence development, supportive organisational cultures and removal of technical barriers. The logic model proved valuable in revealing implementation patterns, implementation gaps, and areas for targeted improvement. Future research should prioritise the validation of the logic model and particularly of the proposed pathways.

## 1 Introduction

Digital technologies, such as barcode scanners and intelligent IT systems, can enhance efficiency, accuracy, and reliability in internal hospital supply chains, while reducing administrative burdens (1), and enabling staff to prioritise patient care (2, 3).

Internal hospital supply chains are characterised by complex workflows, high process volumes, and strong interdependencies between clinical units, logistics, and information systems. Digitalising these processes typically involves the integration of multiple technical components, organisational changes, and user-facing workflows (4). Such interventions therefore constitute complex socio-technical implementations rather than isolated technical upgrades. Their success depends not only on technological functionality but also on user engagement, training, workflow integration, and local implementation conditions like interoperability gaps, data standardisation deficits, heterogeneous sources, and security challenges. Deploying such solutions involves internal divisions and external partners with diverse needs, necessitating tools for planning, communication, and evaluation (5).

Previous studies of health information technologies have reported mixed implementation outcomes. While digital solutions can improve efficiency, transparency, and process quality, implementations often encounter challenges related to technical reliability, limited interoperability, and difficulties integrating new technologies into existing organisational structures (6, 7). In addition, workflow disruptions (6), insufficient training (7), limited user involvement (8), and resistance to organisational change (7, 8) were reported in the literature. Furthermore, discrepancies between system adoption and positive user perceptions had been identified as recurring barriers to successful implementation (6, 9). Consequently, implementation success may vary substantially across organisational contexts, highlighting the need for evaluation approaches that capture not only outcomes but also the underlying mechanisms and contextual factors.

As the literature demonstrates, digital transformation initiatives in healthcare are complex socio-technical interventions whose effects emerge through interactions between technological, organisational, process-related, and user-related factors. Unlike isolated technical interventions, digital systems are embedded within existing workflows, involve multiple stakeholder groups, and evolve over extended implementation periods. Traditional evaluation methods, such as randomised controlled trials (RCTs), often fall short to capture the multifaceted nature of technology implementation in healthcare settings (10), requiring more sophisticated approaches like logic models (11).

Several theoretical frameworks had been proposed to understand the adoption of, implementation, and impact of health information technologies. Models such as the Technology Acceptance Model (TAM) and the Unified Theory of Acceptance and Use of technology (UTAUT) focus on individual determinants of technology acceptance and use (12, 13) and have been widely applied in healthcare settings. Other approaches, including the Information System Success Model (14), the Systems Engineering Initiative for Patient Safety (SEIPS) (15), and the Non-adoption, Abandonment, Scale-up, Spread and Sustainability (NASS) framework (16), additionally consider organisational, technical, and socio-contextual factors influencing implementation outcomes. While these frameworks identify relevant determinants of adoption and success, they generally focus on explaining relationships between constructs or categories of factors rather than explicitly describing how implementation activities are expected to result in outputs and outcomes within a specific organisational context.

Logic models complement these frameworks by providing a structured framework to visualise both the key elements of a programme and the relationships between inputs, activities, outputs, outcomes, and impacts. As such, they are particularly valuable for evaluating complex health technologies while enhancing transparency and clarifying potential causal pathways (17).

Drawing on principles from Implementation Science (18), logic models help explain how implementation strategies, organisational conditions, and contextual influences contribute to implementation results. They support the systematic evaluation of complex interventions by linking resources and activities to measurable outputs and outcomes while accounting for contextual factors that may facilitate or hinder implementation success. By making these relationships explicit, logic models can reduce complexity, support quality assurance, and strengthen the interpretation of implementation results (19).

The components of the logic model include inputs (resources), activities, outputs, outcomes, and long-term impacts. These elements illustrate the connection between the planned work, namely, the essential resources and the activities to be carried out, and the processes and achieved results, such as outputs, outcomes, and impacts (17). Inputs therefore refer to the resources required to implement a project, while activities encompass all behavioural components describing what the programme does with these resources. Outputs are the immediate results of the project activities, showing what has been accomplished through the use of inputs and activities. Outcomes represent the changes or effects achieved among the target groups, describing intended or unintended shifts in the behaviour, knowledge, and skills of the affected employees. Finally, impact refers to the long-term, overarching effects and the overall intended or non-intended benefits of the project (8).

Given the complexity of large-scale digital transformation initiatives in hospital settings, a logic model framework offers a suitable approach for integrating technical, organisational, and user-related perspectives within a single evaluation framework.

Therefore, a logic model framework was employed in the present study to assess the project outputs and outcomes by illustrating the relationships between inputs, activities on outputs and outcomes. This paper will thus explore the following research questions:

RQ0. What are the specific elements of a logic model describing the implementation of a hospital-internal digital supply chain system?
RQ1. To what extent do inputs and activities lead to measurable outputs in hospital supply chain processes?
RQ2. How do these outputs influence key outcomes such as satisfaction and efficiency?
RQ3. Which pathways and interdependencies explain variations in the implementation success across hospital sites?

## 2 Methods

### 2.1 Implementation project and setting

This study presents a long-term evaluation of a digital supply chain system project over more than five years, implemented at Klinikum Region Hannover (KRH), Germany. KRH is a major public hospital network in Lower Saxony, Germany, and comprises ten hospital sites, with 3,000 beds, 100,000 cases annually, and 8,500 employees. TKRH belongs to the group of large regional providers in Germany, a country that ranks third highest regarding number of hospital beds per inhabitants among the OECD countries (20).

The project aimed to transform the internal hospital supply chain into a smart supply chain. Core objectives were to optimise internal supply chain workflows, reduce manual tasks and improve the efficiency, increase the user satisfaction, and improve the accuracy and reliability. The present analysis focuses on material documentation (e.g., documentation of implants, medical devices, sterile goods, implant ID Card), and material request processes (e.g., request of materials, medical devices, vac pumps, rental beds) as shown in Table 1.

**Table 1:** Internal supply chain processes.

Material documentation processes	Material request processes
Material documentation for an implant register Documentation and creation of the implant ID card Documentation of implants Documentation of medical devices/instruments Case-related documentation of materials/medical devices Documentation of sterile goods	7. Request for materials/medical devices 8. Request and deregistration of VAC pumps (or rental) 9. Request for drugs 10. Request and deregistration of rental beds/mattresses (Hill-Rom) 11. Patient-related requests

The technical core of the project was a central intelligent hub integrating the hospital information system and database (HIS) including the materials management system, the electronic health record (EHR) and enabling digital workflows via Android-based mobile devices with barcode scanners. The agile software development, installation, and rollout were managed by GSG mbH in Hannover, Germany.

The project spanned five years and five months (September 2019–January 2025), with continuous scientific monitoring and data collection throughout all phases. Table 2 shows the project schedule with the project and data collection phases.

**Table 2:** Project schedule with project phases and data collection phases.

Timeline	Project phase	Data collection
September 2019–March 2020	Pre-implementation (Baseline)	Kick-off workshops to gain initial understanding of workflows; qualitative interviews
April 2021–September 2021	Pre-implementation (Baseline)	Cross-sectional web survey; interviews and process observations
May 2021–September 2022	Roll-out and pilot phase (material documentation)	Interviews and process observations; first post-implementation survey
June 2022–September 2022	Roll-out and pilot phase (material requests)	First post-implementation survey
August 2024–January 2025	Live mode	Second post-implementation survey
January 2025	Live mode	Qualitative interviews
April 2021–May 2025	Roll-out to live mode (continuous monitoring)	Log data collection for material documentation
June 2022–June 2025	Roll-out to live mode (continuous monitoring)	Log data collection for material requests

### 2.2 Data collection and variable operationalisation

A mixed-methods approach was adopted to capture both the breadth and depth of the implementation process and its outcomes.

#### 2.2.1 Survey prior to implementation

A cross-sectional web survey was employed from April 2021 to September 2021 to capture information, attitudes and expectations from potential users prior to the implementation. Therefore, the survey targeted potential future users. The survey included nine questions per process on the duration of the processes, the satisfaction with the processes, the susceptibility of the processes to errors, expected usefulness of using a scanner, one question on the satisfaction with the project communication, one question on the involvement in the project, four questions on the self-reported information system (IT) affinity, as well as 31 questions on the constructs such as Perceived Usefulness, Effort Expectancy and Continuance Intention. These survey items were informed by the expanded two-stage model of Information System Continuance (21), which also includes the core constructs of the Unified Theory of Acceptance and Use of Technology (13). Participants took part in the survey anonymously and voluntarily. Data was collected across all ten KRH (Klinikum Region Hannover) sites. The target groups of the survey were nurses, surgical and medical technical assistants and other clinical staff (e.g., service assistants, nursing assistants, documentation assistants) in all units of KRH. 310 questionnaires were fully completed.

#### 2.2.2 Post-implementation survey

The post implementation survey was conducted from August 2024 to January 2025. Again, the survey addressed nurses, surgical and medical technical assistants and other clinical staff in all units of KRH who were actively using the scanner after implementation. The questionnaire was designed methodologically consistent with the one of the pre-implementation phases retaining identical items while adding five targeted questions per process about improvements as well as emergent challenges. 148 questionnaires were fully completed.

To assess the comparability of respondents across the survey waves, demographic and professional characteristics were compared between the pre- and post-implementation surveys (Table 3). No significant differences were observed regarding gender (*p* = .860) or clinical departments, e.g., operating room, surgical wards, endoscopy (*p* = .239). However, differences were found in occupational group (*p* < .001), hospital site (*p* = .007), and professional experience (*p* = .004), while age showed a borderline significant difference (*p* = .056). Overall, the two survey samples were broadly comparable regarding demographic characteristics and clinical departments but differed in some professional and organisational characteristics. Therefore, changes observed between survey waves should be interpreted considering potential differences in the two samples.

**Table 3.** Socio-demographic and Professional Characteristics of Participants Before and After Implementation.

**Characteristic**	**Category**	**Pre-Implementation Survey** **May 2021–Sep 2021** ***(n = 310)***	**Post-Implementation Survey** **Aug 2024–Jan 2025** ***(n = 148)***
Gender, %	Male	24.5	26.9
	Female	74.5	72.4
	Diverse	0.7	0.7
Age, years	Median (IQR)	47.3 (19.4)	43.0 (22.7)
Professional experience, years	Median (IQR)	23.3 (39.7)	20.3 (32.7)
Occupational group, %	Nurses	78.7	62.3
	Technical assistants	7.3	25.3
	Others	14.0	12.3

**Note.** Values are percentages unless otherwise indicated. Age and professional experience are presented as median (IQR).

#### 2.2.3 System log data

The degree of utilisation was objectively measured using anonymised system log data systematically extracted from the hospital's digital infrastructure. The recording of the material documentations were extracted from April 2021 to May 2025. The material requests were extracted from June 2022 to June 2025. These log files contained all transactions per month related to the number of documentations and requests, the type of documentation and request (with scanner, without scanner), the location and the organisational unit.

#### 2.2.4 Interviews

Semi-structured interviews were conducted with the three main project leaders. Participants were purposively selected based on their role in project governance and implementation and included representatives responsible for strategic project coordination, operational procurement processes, and technical system implementation. These interviews took place between January and March 2025, lasted between 53 and 179 min and provided qualitative insights into the program's structure, including the identification and description of essential inputs (such as resources and personnel) and activities (such as training and product development). The interviews also yielded information about barriers/facilitators encountered during implementation. These interviews were analysed thematically to contextualise quantitative findings. Additionally, semi-structured interviews were conducted with department heads and two employees at four hospital sites in February and May 2022 focusing on barriers post-implementation on documentation processes.

### 2.3 Logic model establishment and data analysis

The logic model was developed using a retrospective re-engineering approach. First, the model's outputs and outcomes were based on measured variables to ensure a solid empirical foundation. Next, activities and inputs were reconstructed by combining information from qualitative interviews and quantitative data collected over time. This approach enabled a robust triangulation of complementary data sources, including interview data, surveys, log file analyses, and process monitoring to identify gaps in activities (for example, inconsistent training) or inputs (such as unequal resource allocation) that could explain differences in outputs and outcomes. Table 4 shows an overview of the measurement methods used to identify inputs, activities, outputs and outcomes.

**Table 4:** Measurement of logic model components.

Input	Measurement
Funding	Thematic Analysis of qualitative interviews with main projects leaders
Existing Organisation	
Cooperation Partners	
Staff Involvement	
Equipment and materials	
**Activities**	**Measurement**
Communication and Engagement	Thematic Analysis of qualitative interviews with main projects leaders
Training and Support	
Process Analysis and Monitoring	
Data Management	
Product Development	
System Integration	
Infrastructure Setup	
Dashboard Development	
**Output**	**Measurement**
Adoption and Usage System usage after roll-out	Analysis of log files for scanned and not scanned material documentation and material request across all sites
Process re-engineering and workflow standardisation Achieved Improvements, e.g., Optimisation and digitisation of workflows, fewer steps in processes, Minimisation of routine tasks Remaining barriers, e.g., Master data quality, system connectivity, user interactions	Survey Closed question. Respondents were asked: “Which specific improvements, in your opinion, have resulted? (Multiple selection possible)”, e.g., “Better focus on core tasks” “Minimisation of routine tasks through automation” “Minimisation of process steps” “Which specific problems, in your opinion, have resulted? (Multiple selection possible)”, e.g., “Handling of the free-text search” “Connection to the Wifi” “Missing barcodes”
Documentation and data quality More complete and accurate documentation. Enhanced quality of master data.	Surveys Closed questions. Respondents were asked: “Which specific improvements, in your opinion, have resulted? (Multiple selection possible)” “Materials can be documented more consistently (y/n)”, “Easier data entry via scanner (y/n)”
**Outcomes**	**Measurement**
Satisfaction and perceived usefulness Enhancement of satisfaction levels with healthcare processes among stakeholders. Factors influencing user satisfaction: Perceived Usefulness Perceived Ease of Use Satisfaction with the scanner Trust in the system Intention to continue usage Attitudes towards the scanner	Surveys single-statement, closed question measured on a 10-point Likert scale (1 = strongly disagree, 10 = strongly agree): “The process has been improved by scanner support”; “There are enough devices available for the process”; “The process causes a lot of frustration.” “I consider scanner support to be very useful in this process.”
	Surveys 31 closed questions on a 10-point Likert scale (1 = strongly disagree, 10 = strongly agree) based on the two-stage information systems continuance model
Efficiency Time required for material documentation and material request tasks Error reduction	Surveys Comparison of time before and after implementation and frequency of use Single-statement, closed question measured on a 10-point Likert scale (1 = strongly disagree, 10 = strongly agree): “The process is highly prone to errors.”

#### 2.3.1 Data analysis

Data analysis combined quantitative survey data and system-generated process data to evaluate scanner-supported material documentation and material request processes. Analyses of the surveys were conducted in IBM SPSS Statistics 28; the log data analysis was performed in Jupyter Notebooks. All analyses followed a structured approach aligned with the project's logic model.

##### 2.3.1.1 Descriptive analysis of process adoption and usage

To assess scanner uptake over time, monthly counts of documented materials and material requests were aggregated by site and by scanner vs. non-scanner workflows. For trend analyses, 3-period simple moving averages (SMA) were applied to smooth short-term fluctuations. Scan rates were calculated as the proportion of scanner-based events relative to the total of materials documented or requested.

##### 2.3.1.2 Formation of improvement and problem clusters and frequency analysis

Because respondents could select multiple items, improvement and problem variables were grouped into conceptually coherent clusters using binary aggregation (MAX-function in SPSS). This approach classified respondents as belonging to a cluster if they selected any item within that domain. Statements were clustered for both documentation and material request processes, reflecting themes such as efficiency and workload reduction, digitalisation and data quality, or handling and system issues. For each cluster the percentage of responses were computed, indicating the share of all statements that reported at least one issue or improvement within the domain.

#### 2.3.2 Integration into the logic model

To empirically assess logic-model pathways, descriptive Pearson correlation coefficients were calculated to examine linear associations between key implementation variables, user perceptions, and scanner usage metrics. In detail, correlations were analysed a) between participation in project-related training sessions and the perceived level of information about the scanner implementation, b) between satisfaction with involvement in the project and satisfaction with the resulting work processes, and c) between scanner usage rates and workflow activity indicators, including the total number of processed material requests and the total number of documented materials. These analyses aimed to determine whether engagement, communication, and training were associated with higher levels of satisfaction and whether scanner adoption translated into increased workflow activity.

Given the multi-site nature of the implementation, differences between sites were examined using one-way analysis of variance (ANOVA). Dependent variables included perceived ease of use of the scanner system, continuance intention (i.e., the intention to continue using the scanners), attitudes towards scanner use, and overall satisfaction with the digital processes. Effect sizes (*η*² and *ω*²) were calculated to quantify the magnitude of between-site variation. When omnibus tests indicated statistically significant differences, *post hoc* pairwise comparisons were conducted to identify site pairs with meaningful differences. Where applicable, observed site-level differences were interpreted in relation to objective scanner usage rates derived from the log data.

Finally, to assess efficiency-related outcomes and identify remaining barriers, aggregated multiple-response data on reported workflow problems were analysed. These results enabled the identification and mapping of site-specific barriers along the efficiency pathway described in the logic model.

## 3 Results

**Figure 1:**
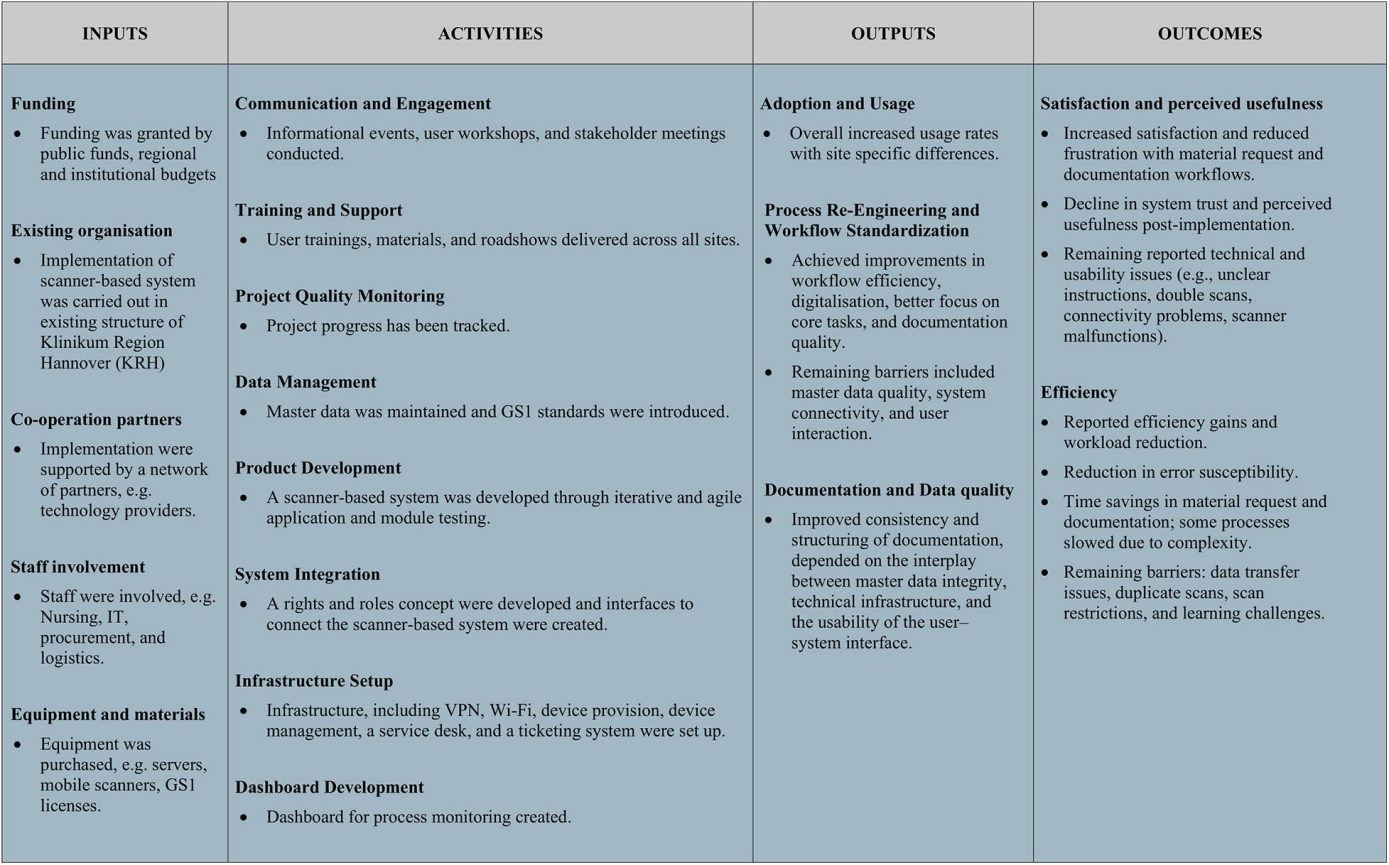
Overview of the elements of the logic model.

The results are presented in alignment with the logic model. To answer research question RQ0, Figure 1 shows the specific elements of a logic model describing the implementation of a hospital internal supply chain along the inputs, activities, outputs and outcomes.

Accordingly, the Results section is organised along these components, starting with inputs and activities, followed by outputs related to adoption, process re-engineering, workflow standardisation, and documentation quality, and subsequently outcomes including satisfaction, perceived usefulness, and efficiency.

### 3.1 Inputs, activities, and their impact on outputs

Inputs and activities in this project comprised the technical, organisational and training related implementation of the internal intelligent supply chain system. Technical deployment was executed at different times across the sites: Material documentation was implemented between May and October 2021. In contrast, the deployment of material request processes followed a staggered schedule starting in June 2022 and ended in 2024.

#### 3.1.1 Inputs

The inputs of the logic model comprised a broad range of resources essential for the implementation of the project. Funding was secured from multiple sources, including public grants, regional and institutional budgets. The project was embedded within the existing organisational structure of Klinikum Region Hannover (KRH) and supported by a network of cooperation partners, represented a diverse mix of organisations, including technology providers specialising in hardware (such as barcode scanners and mobile devices), software and enterprise mobility management solutions, supply chain and data standardisation bodies, as well as companies offering healthcare equipment and consulting services. Staff involvement included teams from IT, procurement, logistics, finance, pharmacy, and nursing (wards, surgery, endoscopy and further units) from all sites, as well as external experts from partner organisations. Necessary equipment and materials included servers, mobile scanners, mobile device management systems, GS1 licenses, and project management software.

#### 3.1.2 Activities

The project activities embraced a diverse package of measures for implementing and introducing the new processes. Communication and engagement measures included organising information events, workshops, and user meetings to inform stakeholders, analyse current workflows, define target processes, and collect user needs and feedback.

Training and support were provided through training courses, roadshows at all hospital locations, and training materials such as online videos, written instructions, and interactive sessions. User Training was conducted hospital-wide with various methods (videos, documents, online meetings). Out of the 133 survey respondents, 58.6% reported having received training, 41.4% had not.

Project quality monitoring was carried out by the KRH procurement department and the software manufacturer GSG mbH to track progress. Data management activities focused on regularly updating and maintaining material master data. Product development comprised the iterative and agile development and testing of the applications and modules. System integration measures covered the development of a rights and roles concept and the development of interfaces to connect the server hub to existing hospital IT systems and external providers. The infrastructure setup combined the establishment of secure networks, device management, the operation of a service desk, and a ticketing system. Finally, an integrated dashboard was developed as a central hub for process monitoring.

#### 3.1.3 Outputs

To assess the direct effects of the implementation on operational workflows, measurable outputs were analysed separately for the two primary process domains affected by the scanner-based system: (1) documentation processes and (2) material request processes. Accordingly, the following sections present quantitative indicators of adoption and usage, workflow re-engineering effects, improvements achieved through scanner-supported work, and scanner-related problems reported by staff answering research question RQ1.

##### 3.1.3.1 Adoption and usage

**Figure 2:**
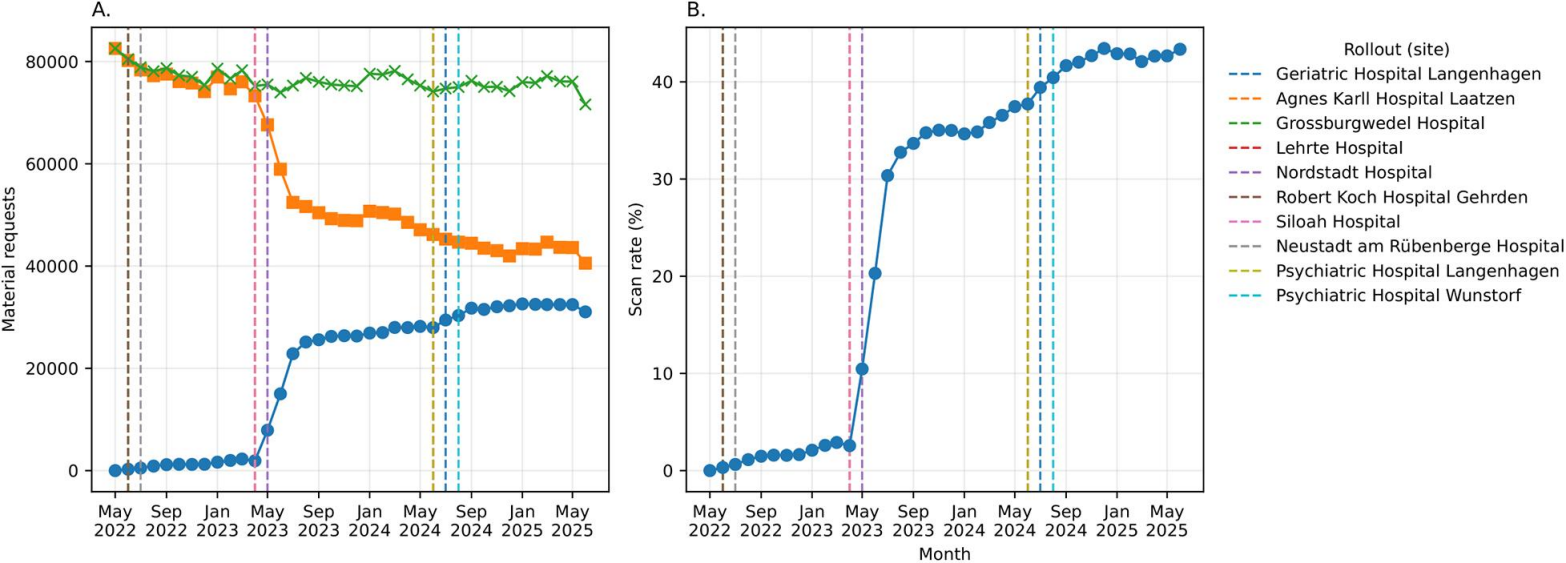
Adoption of scanner-based material request over time (3-period simple moving average), **(A)** material requests in total *(green: total, orange: without scanner-usage, blue: with scanner-usage)* and **(B)** material requests scan rate in % from all material requests.

**Figure 3:**
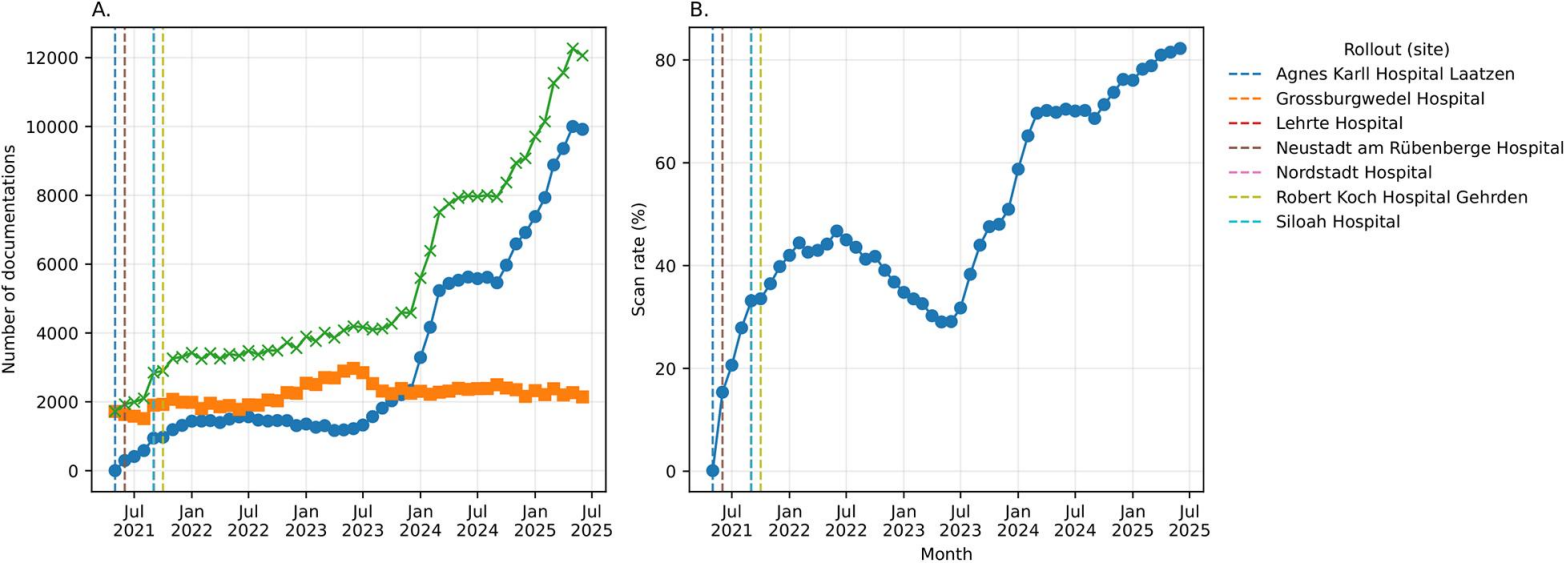
Adoption of scanner-based material documentation over time (3-period simple moving average) **(A)** material documentations in total *(green: total, orange: without scanner-usage, blue: with scanner-usage)* and **(B)** material documentations scan rate in % from all material documentations.

Across the material documentation and material request workflows, scanner-based digital data capture showed a marked increase over time (see Figures 2,3).

Total request volumes remained largely stable over the observation period. However, the distribution between scanner and non-scanner requests shifted markedly: non-scanner requests declined, whereas scanner-based requests increased in the same interval (Figure 2A). The overall scan rate rose sharply following the phased introduction of scanners at the individual hospital sites and stabilised at approximately 45% (Figure 2B).

Scanner-based documentation increased substantially following the stepwise rollout across sites (Figure 3A).

By mid-2025, scanner use accounted for 81% of all documentation events across the network. The overall scan rate for documentation increased steadily (Figure 3B), reflecting a progressive shift from manual to digital documentation workflows.

**Figure 4:**
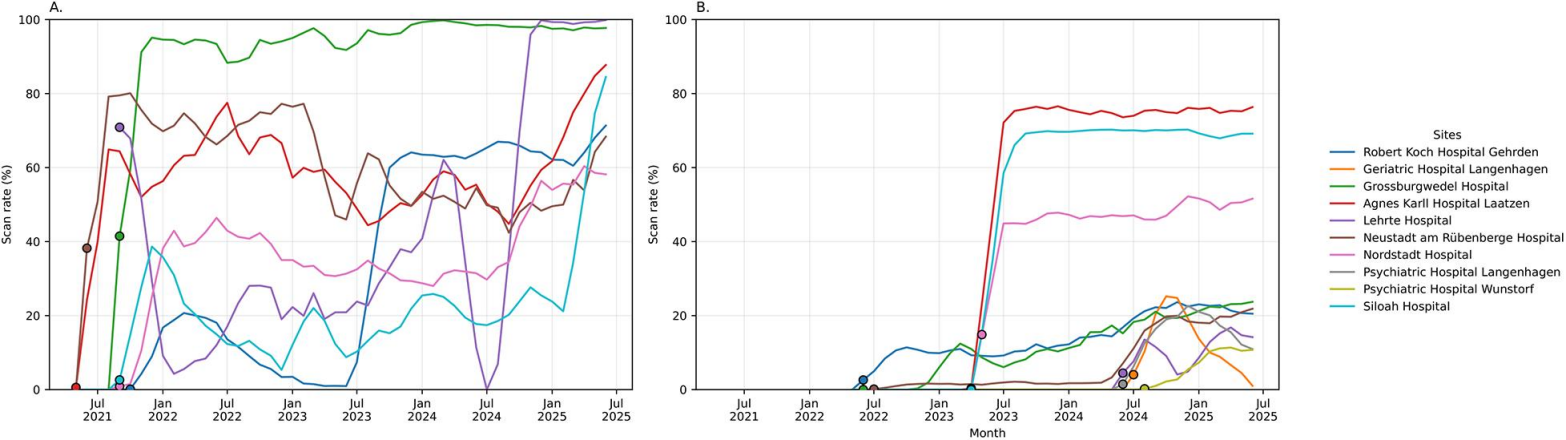
Longitudinal adoption trajectories of scanner-based documentation and material request (**(A)** Scan rate trend for material documentation and **(B)** scan rate trend for material request) across sites (3-period simple moving average).

Adoption and usage curves varied considerably from site to site: For material requests some locations reached scanner shares of 69%–76%, while others remained between 6%–22%. In documentation, uptake was generally higher, with several sites reaching more than 97% scanner usage since 2022, while others increased gradually from lower baseline levels (Figure 4).

##### 3.1.3.2 Process re-engineering and workflow standardisation

Within the logic model, outputs analyses focused on (1) perceived improvements resulting from redesigned workflows, and (2) remaining problems reported by staff. Together, these indicators captured the extent to which scanner-supported processes contributed to workflow standardisation while also revealing persistent operational constraints.

###### 3.1.3.2.1 Perceived improvements following workflow re-engineering

Perceived improvements were reported for both documentation and material request workflows, though exposure to the two process types differed across professional roles. While 21% of respondents performed at least one documentation subprocess, 89% were involved in at least one material request subprocess.

Among the respondents engaged in documentation workflows, 71% (*N* = 22) reported at least one improvement after implementation (Table 5). A total of 70 statements were assigned across four thematic clusters. Reported benefits clustered primarily around workflow efficiency and workload reduction (41.4%), followed by digitalisation and technical facilitation (27.1%). Statements related to documentation quality and consistency accounted for 18.6%, while 12.9% addressed improved focus on core tasks. Staff most frequently highlighted simplified data capture, more structured workflows, and improved consistency of documentation, suggesting that scanner integration supported the standardisation and streamlining of documentation procedures.

**Table 5:** Improvement cluster for documentation processes (*n* = 70 statements by 22 persons).

Improvement cluster	% of statements
1. Workflow efficiency and workload reduction	41.4%
2. Digitalisation and technical facilitation	27.1%
3. Focus on core tasks	12.9%
4. Documentation quality and consistency	18.6%
Total	100.0%

Items represent dichotomous multiple-response variables (coded 1 = mentioned).

For material request workflows, improvements were reported more frequently (Table 6). Among the respondents engaged in request workflows, 84.7% (*N* = 111) reported at least one improvement after implementation. A total of 350 statements were assigned across four thematic clusters. Half of all statements (51.4%) referred to workflow efficiency and workload reduction. Digitalisation and technical facilitation accounted for 26.6% of the statements. Further statements related to documentation quality and consistency represented 11.4% of all responses, while 10.6% referred to an improved focus on core tasks. Taken together, the distribution of improvement categories suggested that workflow re-engineering primarily affected structural aspects of the request process, particularly the replacement of manual steps and reduction of workload and routine task.

**Table 6:** Improvement clusters for material request processes (*n* = 350 statement by 111 participants).

Improvement Cluster	% of statements
1. Workflow efficiency and workload reduction	51.4%
2. Digitalisation and technical facilitation	26.6%
3. Focus on core tasks	10.6%
4. Documentation quality and consistency	11.4%
Total	100.0%

Items represent dichotomous multiple-response variables (coded 1 = mentioned).

###### 3.1.3.2.2 Remaining problems and constraints

Despite substantial improvements, respondents also reported several problem categories affecting both documentation (Table 7) and material request (Table 8) workflows, indicating limits to the effectiveness of process re-engineering.

**Table 7:** Problem cluster for documentation processes (*n* = 81 statements by 22 persons).

Problem cluster	% of statements
1. Master data & barcode/article-related issues	56.8%
2. Technical system & connectivity issues	29.6%
3. User interaction, search & handling issues	13.6%
Total	100.0%

Items represent dichotomous multiple-response variables (coded 1 = mentioned).

**Table 8:** Problem cluster for request processes (*n* = 361 statements by 102 persons).

Problem cluster	% of statements
1. Master data & barcode/article-related issues	52.4%
2. Technical system & connectivity issues	31.9%
3. User handling, search & workflow issues	15.8%
Total	100.0%

Items represent dichotomous multiple-response variables (coded 1 = mentioned).

A total of 81 statements were assigned across three thematic clusters for documentation processes (Table 7). Among the respondents engaged in material documentation, 71.0% (*N* = 22) reported at least one problem after implementation. Challenges most frequently concerned master data and barcode or article-related issues (56.8%), followed by technical system and connectivity problems (29.6%) and user interaction issues such as search functions or scanner handling (13.6%). Together, these findings illustrate that workflow re-engineering introduced substantial procedural improvements but also revealed persistent data- and system-related constraints.

In material request workflows, reported problems showed a comparable pattern but with higher overall occurrence. A total of 361 statements were assigned across three thematic clusters. Among the respondents engaged in material request, 77.9% (*N* = 102) reported at least one problem after implementation (Table 8). The most frequently reported constraints were master data and barcode/article-related problems (52.8%). They included missing or unreadable barcodes, inconsistencies between scanner data and storage modules, unclear product specifications, and challenges related to integrating new products. Technical and system-related problems, such as unstable Wifi-connections, scanner error messages, and delayed data transmission to the hospital information system, were also widespread (31.9%). In addition, user handling, search, and workflow-related issues were commonly reported, including difficulties with free-text search, scanning patient QR codes from computer screens, and ergonomic or usability challenges (15.8%).

Taken together, these findings indicated that while process re-engineering enabled more standardised and digitised workflows for both documentation and material requests, its effectiveness was dependent on data quality, technical infrastructure, and usability of the user-system interface.

##### 3.1.3.3 Documentation and data quality

Within the logic model, documentation and data quality outputs reflected both perceived improvements and persisting constraints across documentation and material request workflows.

For documentation processes, respondents reported more consistent, scanner-based recording of materials and reduced reliance on fragmented or parallel documentation media (18.6% of the statements), indicating a shift towards more standardised data capture (Table 5). In material request workflows, 11.4% of all statements referred to documentation quality and consistency, while 26.6% addressed digitalisation and technical facilitation, covering aspects such as scanner-supported data capture and system-based request routines, suggesting enhanced traceability and completeness of recorded requests (Table 6).

At the same time, documentation quality remained highly dependent on reliable master data and barcode information, 56.8% of all reported documentation problems concerned master data and barcode or article-related issues, including missing or unreadable barcodes, unit mismatches, and unclear product specifications (Table 7). Technical system and connectivity issues accounted for a further 29.6% of documentation-related problems, such as unstable wireless connections, scanner error messages, and delayed data transmission to the hospital information system, further threatened the timeliness and accuracy of documentation. User interaction issues, including difficulties with free-text search, QR code capture from screens, and scanner handling, additionally increased the risk of incomplete or delayed entries represented 13.6% of documentation problem statements and 15.8% of the problems mentioned in material request workflows (Tables 7, 8).

Overall, the pattern shows that scanner-supported workflows improved the consistency and structuring of documentation, but that data quality ultimately depended on the interplay between master data integrity, technical infrastructure, and the usability of the user–system interface.

##### 3.1.3.4 Synthesis of inputs, activities and outputs

Qualitative interviews with project leaders helped to link the observed outputs to the underlying inputs and activities. They emphasised that dedicated funding, cross-site staff involvement, and close cooperation with the software vendor were essential preconditions for sustaining the multi-year roll-out of scanner-based workflows. Communication and engagement activities such as information events, workshops, and user meetings were reported to be crucial for monitoring existing workflows, defining target processes, and aligning expectations across sites. Training and support measures, together with continuous process monitoring and master data maintenance, were perceived as key factors enabling the transition from paper-based to scanner-supported documentation and request processes and as explanations for site-level differences in adoption, satisfaction, and perceived efficiency observed in the quantitative data.

### 3.2 Impact of outputs on key outcomes: satisfaction, perceived usefulness and efficiency

Building on the implementation outputs described above, this section addresses research question RQ2 by examining how scanner-based workflow changes related to key outcome measures. Specifically, we analysed changes in user-reported satisfaction, perceived usefulness, and efficiency, as well as error susceptibility and processing times, in order to assess how the documented outputs of the logic model translated into measurable effects on staff experience and process performance.

#### 3.2.1 Outcomes

##### 3.2.1.1 Satisfaction and perceived usefulness

Satisfaction and perceived usefulness were compared before and after implementation of scanner-based material request workflows (Table 9). Satisfaction with material request processes increased significantly post implementation, accompanied by a significant reduction in reported frustration. In contrast, trust in the system and perceived usefulness showed a moderate but statistically significant decline.

**Table 9:** Pre–post changes in satisfaction, frustration, trust, and perceived usefulness related to material request processes.

Outcome measure	Pre-implementation Mean (SD)	Post-implementation Mean (SD)	Mean difference (95% CI)	*p*-value
Satisfaction	5.0 (2.1)	7.0 (2.4)	−2.04 (−2.55 to −1.53)	<0.001
Frustration	5.9 (2.5)	4.3 (2.7)	1.65 (1.05 to 2.24)	<0.001
Trust in the system	7.8 (2.1)	6.4 (2.7)	1.36 (0.80 to 1.93)	<0.001
Perceived usefulness	8.1 (1.9)	6.8 (2.7)	1.34 (0.80 to 1.87)	<0.001

Measured on a 10-point Likert Scale. Higher values indicate a higher level of the respective metric. *p*-value: Two-sided independent-samples *t*-test.

User-reported experience measures for documentation workflows were compared before and after the implementation of scanner-based documentation processes (Table 10). Satisfaction with documentation processes increased significantly post implementation, while frustration related to documentation showed a non-significant tendency to decrease. In contrast, trust in the system and perceived usefulness of the documentation functions showed a moderate but statistically significant decline.

**Table 10:** Pre–post changes in satisfaction, frustration, trust, and perceived usefulness related to material documentation processes.

Outcome measure	Pre-implementation mean (SD)	Post-implementation mean (SD)	Mean difference (95% CI)	*p*-value
Satisfaction	5.0 (2.2)	7.0 (2.6)	−2.00 (−3.18 to −0.83)	<0.001
Frustration	5.3 (2.9)	4.2 (3.0)	1.10 (−0.30 to 2.50)	0.105
Trust in the system	7.9 (1.9)	6.8 (2.5)	1.10 (0.03 to 2.18)	0.044
Perceived usefulness	8.4 (1.8)	6.8 (2.5)	1.58 (0.56 to 2.60)	0.003

Measured on a 10-point Likert Scale. Higher values indicate a higher level of the respective metric. *p*-value: Two-sided independent-samples *t*-test.

Interviews highlighted drawbacks in perceived usefulness related to unclear barcode scanning instructions, double scans, slow or unreliable data transmission to the hospital information system, scanner malfunctions, and limited scanning of non-sterile implants.

##### 3.2.1.2 Efficiency

Across processes, efficiency-related effects were frequently reported by participants. Overall, 51.4% of all coded statements in material request processes (Table 6) and 41.4% of all coded mentions in documentation processes (Table 5) referred to at least one aspect related to efficiency and workload reduction.

For material request processes, error susceptibility decreased significantly after implementation (pre: 5.2 ± 2.33, post: 3.8 ± 2.50), with a mean difference of 1.38 points (CI: 0.87 to 1.91), indicating a clearly lower perceived vulnerability to errors in scanner-supported material request workflows. Error susceptibility for documentation processes was slightly lower post implementation (pre: 5.0 ± 2.84, post: 4.4 ± 3.00; mean difference 0.60, CI: −0.67 to 1.86).

Time savings varied: documentation time dropped by 9.1% (median 5.5 min pre- to 5.0 min post-implementation). Material request processes showed greater savings (50%), with median times dropping from 10.0 to 5.0 min. Some drug request processes, however, increased in time (median increased from 10.0 to 25.0 min), indicating variable efficiency based on process complexity. Interview data indicated that slow or faulty data transfer, duplicated scans, media breaks due to scan restrictions (sterile vs. non-sterile implants), and challenges when familiarising with the new system. In addition, respondents reported that scanner-based drug requests often required additional approval steps in the ScanProCare scanner application and the central materials management system, resulting in more process steps than before implementation. Users also described incomplete workflow integration, including non-scannable medications, missing QR-Codes, and special requests that could not be efficiently processed using the scanner.

### 3.3 Evidence related to the proposed pathways and differences across sites

**Figure 5:**
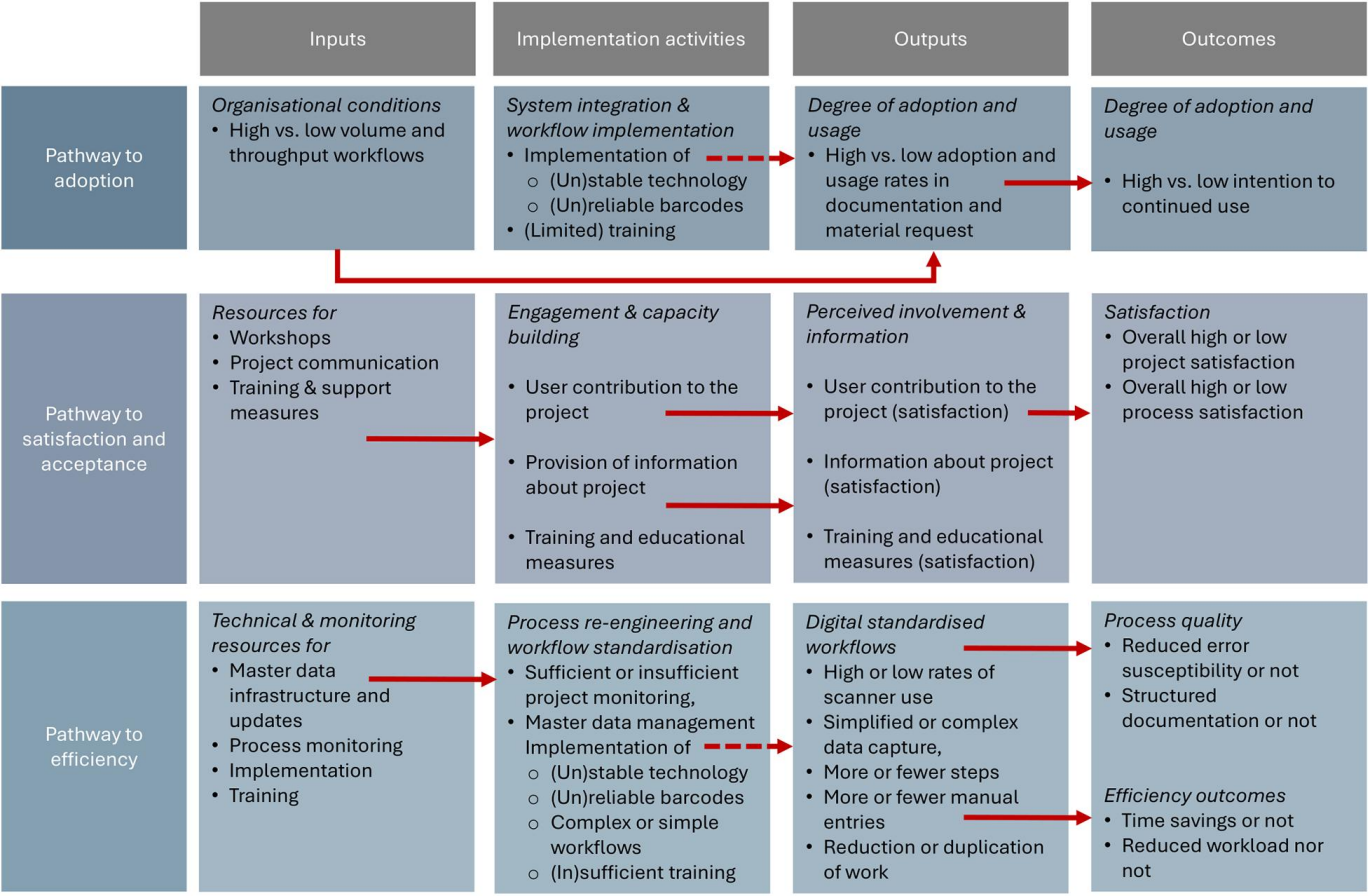
Logic model pathways (solid line: association, dashed line: direction of association conditional on context).

The logic model proposes three interrelated pathways through which the digital implementation may influence outcomes, addressing research question RQ3 (Figure 5): a pathway to adoption and usage, a pathway to satisfaction and acceptance and a pathway to efficiency.

#### 3.3.1 Pathway-consistent evidence for adoption and usage

The pathway to adoption and usage in the logic model describes how technical and organisational inputs, together with system development and integration activities, were associated with varying degrees of implementation, reflected in adoption and actual system usage.

The empirical findings provide evidence consistent with the proposed adoption and usage pathway, showing that higher scanner usage rates were positively associated with operational activity. Specifically, scanner usage correlated moderately with material request volumes (*r* = .365) and with the number of documented materials (*r* = .364), indicating that scanners were used more often in cases of high throughput workflows.

In addition, significant site-level differences were observed in acceptance-related outcomes closely linked to continued system usage. One-way ANOVA revealed significant between-site variation in continuance intention [F(9,125) = 2.04, *p* = .041, *η*² = .13, *ω*² = .07] and attitudes towards system use [F(9,132) = 2.71, *p* = .006, *η*² = .16, *ω*² = .10]. *Post hoc* comparisons showed that differences in continuance intention between sites corresponded with substantial differences in observed scan rates, such as 50% vs. 6%, linking subjective intentions to objective adoption patterns.

Across sites, qualitative feedback pointed to substantial variation in the local conditions under which scanner-based workflows were implemented. Units with frequent scanner and barcode failures, unstable Wifi, complex or duplicative material request steps, and limited training (e.g., Nordstadt, Großburgwedel, the psychiatric units) reported low or selective scanner use and a continued reliance on manual processes, whereas sites with more reliable scanner functionality and clearer procedures (e.g., the pilot site) described more positive experiences and broader routine use. These qualitative patterns were consistent with the significant between-site differences in overall satisfaction observed across sites [F(8,131) = 3.92, *p* < .001, *η*² = .221, *ω*² = .161]. These findings corroborate the assumption that adoption and usage processes were shaped by site-specific implementation contexts rather than following a uniform trajectory.

To further explore site-level differences, site-specific adoption levels derived from scanner utilisation data were linked to post-implementation user perceptions. Exploratory correlation analyses revealed weak negative associations between adoption level and performance expectancy (*r* = -.18, *p* = .033), social influence (*r* = -.19, *p* = .035), and attitudes towards system use (*r* = -.21, *p* = .014). Similar trends were observed for trust (*r* = -.17, *p* = .054), while no significant associations were found for effort expectancy, facilitating conditions, self-efficacy, or continuance intention. These findings indicate that sites with higher level of adoption were not necessarily accompanied by more favourable user perceptions.

#### 3.3.2 Pathway-consistent evidence for satisfaction and acceptance

In the logic model, a proposed pathway linked satisfaction with implementation processes to satisfaction with user-centred participation. Consistent with the assumptions underlying this pathway, a significant positive association was observed between satisfaction with staff contributions to the project and overall process satisfaction for both material request and documentation processes. In request processes, this association was moderate (*r* = .362, *n* = 92), while a stronger association was found for documentation processes (*r* = .465, *n* = 23). Another proposed relationship concerned satisfaction with educational measures was connected to the level of information about the project. This meant that the perceived amount of information about the project, reflecting communication and engagement activities, were positively associated with the satisfaction with training and support measures. For material request processes, this association was moderate (*r* = .465, *n* = 71). A similar association was observed for documentation processes (*r* = .427, *n* = 15).

Site-level analyses demonstrating that contextual conditions influence acceptance-related outcomes. In KRH Psychiatric Hospital Langenhagen, high perceived ease of use (mean 7.7) coincided with structural barriers such as limited scanner access, missing user profiles, absent training, and delayed IT support. This likely constrained actual use despite favourable usability ratings and points to problems of perceived usefulness rather than ease of use. In contrast, KRH Klinikum Agnes Karll Laatzen showed lower ease-of-use ratings (mean 3.8) but more established routine integration, with feedback centring on concrete technical issues. These circumstances may explain higher scan rates despite usability concerns. This difference illustrates how local implementation conditions can moderate the translation of user involvement and information into satisfaction and acceptance outcomes.

Taken together, these findings provide evidence consistent with the proposed pathway depicted in the logic model, in which communication, training, and user involvement are linked to satisfaction and acceptance outcomes, while also highlighting that these outcomes may vary across sites depending on local conditions.

#### 3.3.3 Pathway-consistent evidence for efficiency

Within the logic model, a proposed efficiency pathway linked technical and organisational inputs with project quality monitoring, data management, and system integration activities. These activities enabled process re-engineering and workflow standardisation as immediate outputs.

The observed efficiency-related outcomes across both documentation and material request processes provide evidence consistent with the assumptions underlying this proposed pathway. For material requests, 51.4% of all reported improvements and 41.4% of improvements in documentation workflows referred to workflow efficiency and workload reduction (Tables 5, 6), while error susceptibility scores for request processes decreased by 1.38 points on average, with the 95% confidence interval ranging from 0.87 to 1.91 (see Section 3.2.1.2). These effects were reflected in quantitative indicators as well as in qualitative reports of improved workflow efficiency and reduced workload. Sites such as Lehrte, Neustadt, Langenhagen, which showed comparatively high scores on perceived process efficiency and overall satisfaction, also reported simplified data capture, more structured ordering steps, and fewer manual entries in scanner-supported workflows. In contrast, sites like Nordstadt, Großburgwedel, and Robert Koch Gehrden, which scored below the overall mean on efficiency- and satisfaction-related scales, described frequent scanner or barcode failures, time-consuming manual data entry, duplicate orders and complex ordering mechanisms, and continued reliance on manual materials management processes. Across sites, respondents additionally reported barriers such as slow or unstable scanner applications, repeated scans due to unregistered products, restrictions on scanning certain implants, weak Wifi, and insufficient or outdated training. As a result, the translation of process re-engineering outputs into efficiency gains varied across locations, with some workflows benefiting from noticeable time savings and reduced workload, while others experienced only limited or even negative efficiency effects. Overall, these findings demonstrate variability in implementation outcomes across the hospital sites, reflecting heterogeneous implementation conditions within the hospital network.

Taken together, the results across the three proposed pathways indicated that the implementation did not follow a uniform or linear trajectory across sites. While adoption and observed usage were closely linked to operational activity, the translation of system use into acceptance, satisfaction, and efficiency gains was strongly shaped by the local implementation conditions. The logic model pathways (Figure 5) therefore reflected an empirically informed understanding of the digital implementation as a context-dependent process, in which technical reliability, workflow integration, and organisational support moderated how implementation activities were transformed into sustained use, user acceptance, and efficiency outcomes.

## 4 Discussion

The present study developed a logical model encompassing inputs, activities, outputs and outcomes based on the evaluation of a hospital internal supply chain management project. The technical intervention embraced implementing barcode scanners and an intelligent workflow hub to improve the internal supply chain processes. Scanner use for material documentation reached up to 99% as of mid-2025, however strongly varying across sites particularly for material request. User satisfaction improved significantly post-implementation, and frustration decreased. Efficiency gains were observed through reduced process steps and fewer errors. However, trust in the system and perceived usefulness declined. This pattern is noteworthy because it illustrates that increased system utilisation and process improvements do not necessarily translate into more favourable user perceptions. There were significant site differences in satisfaction and intention to continue. Technical barriers, such as unit mismatches and barcode reading issues, were especially frequent at sites with low scanner usage. The evaluation confirms progress along the three key implementation pathways identified in the logic model: usage and adoption, satisfaction and acceptance, and efficiency.

### 4.1 Adoption and usage pathway: interpretation of findings

Positive correlations between volume (number of requests or documentations) and scanner usage suggest that higher process volumes encourage adoption. Technical and organisational challenges reported in interviews, such as screen glare, familiarisation issues, hard-to-read barcodes, limited scanner access for temporary staff could interfere with usage. Similar patterns have been described in technology acceptance research, where perceived usefulness, ease of use, and fit with local workflow shape the initial adoption of clinical information systems (22).

Interpreted within a logic model framework, the findings are consistent with the proposed adoption and usage pathway linking behavioural engagement, user perceptions, and continued use. The positive associations between scanner usage rate and operational indicators such as continual material requests and documentation frequency point to a connection between a need for digital assistance and routine integration into everyday work processes. Site-level differences in attitudes towards use, continuance intention, and satisfaction align with observed differences in actual usage rates, indicating that behavioural uptake and user perceptions co-occur within implementation contexts. Satisfaction appears to function as a reinforcing element within the proposed pathway, potentially supporting the stabilisation of use over time. The consistency of findings across objective usage data, site-level differences in attitudes and continuance intention, and qualitative descriptions of local implementation conditions does not prove causality but provides pathway-consistent evidence for the proposed adoption-and-usage pathway (23). Together, these findings suggest that implementation context was reflected in differences in routine system use as well as acceptance-related outcomes across sites.

Beyond the hypothesised pathway, additional exploratory analyses examined whether sites with higher levels of scanner adoption also reported more favourable user perceptions. These analyses revealed that higher site-level adoption was not necessarily associated with more favourable user perceptions. Weak negative correlations were observed between adoption level and performance expectancy, social influence, and attitudes towards system use. This finding suggests that sustained system use and positive user perceptions represent related but distinct dimensions of implementation success. While scanner-supported workflows became increasingly integrated into routine practice, users in highly digitalised environments may also have become more aware of workflow constraints, technical limitations, and implementation challenges (24, 25). This may have resulted in more critical evaluation of the system. Another explanation which is supported in the literature (26) may be that actual system usage and user acceptance may diverge in organisational settings were digital workflows become embedded in routine practice and usage is mandatory. So, in contrast to implicit assumptions that higher usage rates come along with higher acceptance rates, our findings point to a more nuanced interpretation. High acceptance rates may reflect the obligation to use the system as well as they provide the opportunity to go into the depth of the system and detect inconsistencies and flaws.

### 4.2 Satisfaction and acceptance pathway: interpretation of findings

Post-implementation, process satisfaction improved significantly while frustration decreased. User training was strongly linked to feeling informed about the project and increased satisfaction. Nonetheless, trust in the system and perceived usefulness declined, indicating that adoption does not automatically translate to full acceptance. This interpretation is further supported by exploratory analyses showing that higher site-level adoption was not associated with more positive user perceptions. Instead, weak negative associations were observed between adoption levels and several acceptance-related constructs. This suggests that routine use and user acceptance may evolve differently over time.

Site differences in ease of use and satisfaction illustrate the influence of local context and user experience. For example, at sites where users reported insufficient training, unclear scanning rules and frequent double scans, satisfaction scores remained closer to pre-implementation levels despite objectively increased usage, highlighting how local experience moderates the satisfaction pathway. User interviews revealed recurring problems, such as unclear scanning instructions and frequent double scans, that could contribute to decreased trust and perceived usefulness.

Within a logic model framework, the findings are consistent with a proposed a pathway to satisfaction in which staff involvement and the provision of information are associated with more positive views of the processes and implementation measures, consistent with studies showing that user participation and engagement strategies such as early involvement, clear communication, and tailored training improve acceptance of hospital information systems (27, 28). The consistent associations between satisfaction with staff contributions and overall process satisfaction across both process types suggest that perceived meaningful participation is closely linked to how the resulting processes are experienced. Within the logic model, staff involvement may represent one mechanism connecting process design activities to satisfaction-related outcomes.

Similarly, the association between perceived project-related information and satisfaction with training activities points to the importance of getting informed within the satisfaction pathway. Transparent communication and adequate information provision may enable staff to better understand and engage with implementation measures, thereby enhancing satisfaction. Lee et al. (29) emphasises that effective technology training in healthcare should combine practical, scenario-based learning, role specific training materials, phased learning approaches, and ongoing technical support.

The convergence of associations across multiple satisfaction-related constructs supports the plausibility of participation- and information-based pathways to satisfaction. These pathways complement adoption- and usage-related pathways by highlighting satisfaction as a relevant outcome and potential reinforcing factor within the implementation processes.

### 4.3 Efficiency pathway: interpretation of findings

The findings suggest that efficiency gains associated with scanner-supported processes can be understood within a proposed pathway of workflow restructuring enabled by digitalisation. Reductions in error susceptibility and time savings in documentation and material request processes indicate that replacing manual steps with scanner-based activities can streamline workflows and reduce workload. Qualitative evidence supports this interpretation, with respondents frequently reporting efficiency and workload reduction, digital facilitation, and improved focus on core tasks as key benefits.

However, the efficiency pathway appeared to be highly context dependent. Technical and operational barriers, such as slow or faulty data transfer, duplicated scanning activities, media breaks caused by restrictions on scanning sterile vs. non-sterile implants, and insufficient familiarisation with the technology disrupted the translation of digital activities into efficiency outcomes. This explains why efficiency gains were uneven across process types and, in some cases, reversed, as observed in increased processing times for more complex drug request processes. Qualitative feedback suggests that these increases were not solely attributable to technical problems, but also to workflow design constraints. Several users reported that scanner-based drug requests still required additional approval steps in the ScanProCare scanner application and the central materials management system, resulting in more process steps than before implementation. Respondents further described missing QR-Codes, non-scannable medications, and limitations in processing special drug requests, which reduced the potential efficiency gains of scanner-supported workflows. Rather than following a linear cause-effect relationship, the pathway to efficiency appears to be moderated by implementation quality, technical reliability, and process complexity.

Taken together, these findings highlight that efficiency should not be understood as an automatic consequence of digitalisation. Instead, efficiency gains materialise when digital tools are well integrated into existing workflows and supported by adequate technical infrastructure and training. Our data confirmed that process standardisation and scanner-supported workflows can substantially reduce errors susceptibility and workload in routine documentation and material request processes. At the same time, the example of drug request processes demonstrated that digitalisation may also introduce new inefficiencies. These findings are consistent with studies on barcode medication administration and other digital hospital systems, which report that scanning technologies can streamline workflows and reduce errors, but that technical problems, workflow misfit and workarounds often offset expected efficiency gains (30, 31). The proposed efficiency pathway is therefore best conceptualised as conditional and non-linear, with both enabling and constraining mechanisms shaping its outcomes.

### 4.4 Interrelationship between pathways

Synthesising the findings across the proposed pathways suggests a logic model in which efficiency, satisfaction, and adoption and usage are interrelated rather than independent outcomes. The findings related to the pathway to adoption and usage suggest that initial behavioural engagement with the scanner is associated with favourable attitudes, continuance intention, and satisfaction, forming a reinforcing loop that stabilises use over time. The proposed pathway to satisfaction highlights the role of staff involvement and information provision as mechanisms linking implementation activities to a positive perception of the processes and training measures.

The proposed pathway to efficiency intersects with both pathways. Perceived efficiency and workload reduction can reinforce positive attitudes and satisfaction, thereby supporting continued use. Conversely, technical problems and workflow disruptions not only attenuate efficiency gains but may also undermine satisfaction and weaken adoption. Examples such as drug request process illustrate this interaction, where additional approval requirements and incomplete workflow integration reduced efficiency while simultaneously contributing to more critical user perceptions. Together, the findings are consistent with interacting pathways illustrate that implementation outcomes unfold through interacting mechanisms, shaped by contextual and technical moderators.

The pathways underline that digital health initiatives require not only technology but also ongoing organisational support and user involvement to achieve their full potential. Although this study focused on an internal hospital supply chain, the identified pathways from adoption via satisfaction to efficiency mirror mechanisms described in the digital health and supply chain literature in general (32). It emphasises that digital tools only realise their efficiency potential when they are well integrated into clinical workflows and organisational structures, and when users experience them as usable and supportive of their work.

### 4.5 Methodological challenges of building pathways in logic models

These interacting pathways also illustrate methodological challenges of specifying and empirically examining mechanisms within logic models. The literature reveals a persistent gap between the conceptual role of (causal) pathways in logic models and their actual application. While logic models are frequently used to structure and communicate interventions, explicit specification of how inputs and activities lead to outcomes is uncommon. Many studies present logic models that list inputs, activities, outputs, and outcomes without articulating links between them (e.g., 19, 33–36). As a result, the underlying assumptions about mechanisms of change remain largely implicit. Some publications include isolated causal statements, such as the assumption that regular surveys improve professional performance (37) or retrospective interpretations of unexpected program effects (38), but they are not embedded in a coherent set and network of pathways within the models themselves.

A notable inconsistency emerges between normative claims and modelling practice. Several authors identify clear causal relationships as a key quality criterion of logic models (e.g., 39, 40), however, do not explicitly link inputs to outcomes or operationalise proposed mechanisms of change.

Only few contributions attempt to make (causal) pathways explicit. Mills et al. (41) illustrate a “Type 3 logic model” that connects intervention components through arrows, although the empirical basis of these pathways remains unclear. More detailed examples are provided by Bindel et al. (42), drawing on Allende-Richter et al. (43), who present cause-effect chains related to patient self-management. Process-oriented logic models have been proposed as particularly suitable for representing linear and non-linear causal pathways (44, 45), but existing applications often remain general with respect to the underlying mechanisms.

Several authors explicitly identify unclear or missing pathways as a limitation of logic model–based approaches (e.g., 46, 47). This has contributed to the growing interest in theory of change approaches, which place greater emphasis on articulating (causal) assumptions (48, 49). However, even in this literature, pathways are frequently assumed rather than systematically specified or empirically examined.

Overall, (causal) pathways are widely acknowledged as central to high-quality logic models but are rarely made explicit in practice. Strengthening logic models as analytical tools requires a more consistent and empirical foundation of the assumptions and their presentation as explicit, testable hypotheses about mechanisms of change.

### 4.6 Limitations

The application of the logic model framework provided a structured approach to map pathways from inputs and activities to outputs and outcomes. However, some outcomes, particularly long-term impacts, could not yet be assessed within the observation period.

A further methodological consideration relates to the retrospective development of the logic model. Logic models may serve either implementation planning or programme evaluation purposes. In the present study, the model was developed as an evaluation framework and therefore synthesised findings from formative and summative evaluation activities conducted throughout the five-year implementation period. While this retrospective perspective may increase the risk that pathways appear more coherent in light of known outcomes, it also enabled the integration of multiple data sources and stakeholder perspectives. Consequently, the identified pathways should be interpreted as empirically supported associations that provide a foundation for hypothetical causal pathways rather than prospectively specified causal hypotheses that had been tested.

The pathways presented in this study are derived from the underlying logic model and were empirically examined using correlation-based analyses. This analytical approach was chosen considering the available data structure, sample sizes within specific process groups, and the multi-site implementation context. While correlation analyses allow for the identification of statistically meaningful associations between implementation activities, user perceptions, and usage outcomes, they do not permit the estimation of complex, multivariate causal structures or the testing of mediation effects. More advanced modelling approaches, such as structural equation modelling, would require larger and more homogeneous samples, longitudinal measurements at the individual level, and stronger assumptions regarding model specification. Furthermore, context-dependent variability of the outputs and outcomes among the different sites resulted in moderate correlations. Consequently, the identified pathways should be interpreted as empirically supported associations aligned with the logic model rather than as fully specified causal mechanisms.

Although respondent characteristics were compared across survey waves, significant differences in professional role, site distribution, and professional experience were observed. Consequently, some of the observed differences may reflect changes in the composition of the samples rather than implementation effects alone.

Furthermore, the multi-site nature of the implementation introduces additional context variability. The study comprised ten hospital sites with substantial differences in implementation progress, scanner utilisation, and user perceptions. While site-specific analyses and qualitative findings provided valuable insights into local implementation conditions, the available sample sizes within individual hospital sites did not permit more advanced analyses accounting for clustering effects (e.g., multilevel modelling) or site-level causal mechanisms. Consequently, the identified pathways should be interpreted as reflecting overall implementation patterns across the hospital network rather than site specific causal relationships.

Finally, the study was conducted within a single hospital network in Germany. Although the multi-site design and long implementation period enhance the practical relevance of the findings, transferability to other healthcare systems, organisational settings, or digital transformation initiatives should be considered with caution.

### 4.7 Practical implications and further research

The findings suggest that successful digital transformation of hospital supply chains requires more than the technical deployment of digital tools. First, implementation strategies should place particular emphasis on continuous communication, user involvement, and training activities. The observed associations between information levels, training satisfaction, user participation, and process satisfaction indicate that the implementation success depends not only on system functionality but also on how staff are engaged throughout the transformation process.

Second, organisations should recognise that adoption, acceptance, and efficiency represent distinct dimensions of implementation success. Increasing system utilisation alone does not necessarily translate into positive user perceptions. Consequently, implementation evaluations should combine objective usage indicators with measures of user acceptance, satisfaction, and workflow integration.

Third, the considerable variation observed across hospital sights highlights the importance of local implementation conditions. Technical reliability, scanner availability, workflow integration, user support, and training structures influenced how implementation activities translated adoption, satisfaction, and efficiency outcomes. Healthcare organisation should therefore avoid assuming uniform implementation effects across departments or sites and instead monitor local barriers and facilitators throughout the implementation process.

Finally, the findings demonstrate that different process types may benefit differently from digitalisation. While many workflows showed improvements in efficiency and standardisation, more complex processes, such as medication requests, remained vulnerable to workflow disruptions and additional administrative steps. Future implementations should therefore pay particular attention to process-specific requirements and potentially unintended consequences of workflow redesign.

Future research should further investigate how implementation context influences the relationship between adoption, acceptance, and efficiency outcomes. Longitudinal mixed-methods studies and comparative analyses across healthcare organisations may help better understand how digital transformation pathways evolve over time and under different organisational conditions.

## 5 Conclusion

The findings of this study suggest that successful digital transformation of hospital supply chains can be understood through three interrelated implementation pathways, namely adoption and usage, satisfaction and acceptance, and efficiency. Adoption and usage improved notably, especially for material documentation, however, site-specific barriers and limited scanner access restrained full adoption. Satisfaction increased and frustration declined, but trust and perceived usefulness decreased, indicating the need for reliable technology and tailored user support to maintain acceptance. Efficiency gains through fewer process steps, error reductions, and time savings were achieved, but technical issues and data challenges limited their full potential.

Taken together, these findings highlight, that adoption, satisfaction and efficiency represent interconnected but distinct dimensions of implementation success whose relationships appear to be shaped by organisational and technical context. The proposed logic model provides an empirically informed framework for structuring the context, output and outcomes and identified critical factors along the three proposed pathways to future improvements.

## Data Availability

The raw data supporting the conclusions of this article will be made available by the authors, without undue reservation. Requests to access the datasets should be directed to the corresponding author.
